# Dementia patients in palliative care according to data from the German National Hospice and Palliative Care Register (2009–2021)

**DOI:** 10.1186/s12904-024-01509-0

**Published:** 2024-07-25

**Authors:** Carolin Donath, Christoph Ostgathe, Maria Heckel

**Affiliations:** 1 Center for Health Services Research in Medicine, Department of Psychiatry and Psychotherapy, University Hospital Erlangen, Friedrich-Alexander-Universität Erlangen-Nürnberg, Schwabachanlage 6, 91054 Erlangen, Germany; 2 Department of Palliative Medicine & Comprehensive Cancer Center, CCC Erlangen – EMN, University Hospital Erlangen, Friedrich-Alexander-Universität Erlangen-Nürnberg, Krankenhausstraße 12, 91054 Erlangen, Germany

**Keywords:** Dementia, Cognition disorders, Palliative Care, Palliative Medicine, Health Services Research, Health services accessibility, Clinical coding, Documentation

## Abstract

**Background:**

People with dementia are less in focus of palliative care research than other patient groups even though the awareness of their palliative and end-of-life care needs is rising. Empirical data analyses on people with dementia in palliative care services are lacking.

**Aim:**

To explore the prevalence of dementia diagnoses as per the ICD criteria among users of various palliative care settings and to compare use of palliative services, care pathways, and outcomes in people with and without a dementia diagnosis.

**Design:**

We conducted retrospective analysis of dementia diagnoses as per ICD (F00-F03/G30) in the German National Hospice and Palliative Care Register between 2009 and 2021. The analysis used methods of descriptive and inferential statistics, including the Bonferroni correction for alpha error inflation.

**Setting/participants:**

We limited the analysis to the subsample of people aged over 64.

**Results:**

The prevalence of dementia in the different settings of palliative care was lower than in the age-comparable population: Of the 69,116 data sets included in the analysis, a small minority (3.3%) was coded with dementia as the principal diagnosis. Among patients on inpatient palliative care wards, 0.8% (148 of 19,161) had a dementia diagnosis, as did 2.2% (52 of 2,380) of those under hospital palliative care support teams and 4.3% (2,014 of 46,803) of those receiving specialized palliative care at home.

**Conclusions:**

The records of the German National Hospice and Palliative Care Register suggest that the prevalence of dementia is lower than one might expect from general population data, though numbers are in line with international studies on proportion of dementia patients receiving palliative care. Future research could usefully examine whether this discrepancy stems either from omissions in coding dementia as patients’ principal diagnosis respectively from lapses in documentation of a dementia diagnosis previously made, or from barriers to accessing palliative care services or even displays being excluded from palliative care when trying to access it.

**Trial registration:**

No registration.

**Supplementary Information:**

The online version contains supplementary material available at 10.1186/s12904-024-01509-0.

## Background

To date, there is no cure for dementia; therefore, it remains a life-limiting disease [1]. The trajectory of symptom burden leads to an undeniable need for palliative care to maintain quality of life and plan for future care [2–6]. Nevertheless, there is currently a lack of both evidence-based recommendations for the palliative end-of-life care of people with dementia and empirical data on the care they receive in the palliative care context [1, 4, 7, 8]. At the end of 2022, the German Network for Health Services Research published a memorandum on research relating to the last year of life, highlighting the need for people with multimorbidity and dementia to have access to palliative care and the current dearth of high-quality quantitative and qualitative research in this area [9]. One reason for this latter may be the historical development of palliative care, which initially focused on cancers [3, 10], eventually extending its breadth to other diseases in the wake of the updated World Health Organization statement on palliative care [11].

A cross-sectional, population-based study in Spain, points to dementia patients as the second largest group of people with advanced long-term conditions and in need of palliative care (at 23.4% of the population, second in size to the group of cancer patients) [12]. A longitudinal multicenter study of people with dementia, published in 2020, analyzed the locations in which deaths took place, and found that none of the dementia patients among its sample had died in a specialized palliative care context such as an inpatient hospice or an inpatient palliative care ward, or while in receipt of specialized palliative home care [7]. This might indicate that the coding of dementia may not reflect the condition’s actually occurring extent; according to the literature, more than half of older adults treated in acute care hospitals have a degree of cognitive impairment [13], yet only 37% of people with dementia treated in acute care hospitals have this diagnosis officially on their medical records [14].

Noting these gaps in existing work, the aim of the research detailed in this paper was to investigate how frequently people with documented dementia diagnoses are registered (and thus treated) in the various settings providing palliative care.

### Aims

- Analysis of the prevalence with which a national palliative care register records documented principal dementia diagnoses as per the ICD criteria;
- Comparison of how frequently, according to this register, people with a documented ICD-coded dementia diagnosis receive palliative care within each type of hospice and palliative care service;
- Comparison of palliative care setting types, care pathways, and outcomes among people with and without a documented ICD-coded dementia diagnosis whose cases this register records.

## Methods

### Design

The research was a registry-based study with analysis of secondary data. We carried out a retrospective analysis of cases with (and without) recorded ICD-coded dementia diagnoses (F00-F03/G30) in the 2009 to 2021 datasets of the German National Hospice and Palliative Care Register (Nationales Hospiz- und Palliativregister, NHPR). This study meets all five of the CODE-EHR minimum framework standards for the use of structured healthcare data in clinical research [15].

### Setting/participants

The German National Hospice and Palliative Care Register is a voluntary register to which hospice and palliative care services, such as inpatient palliative care wards, inpatient hospices, and specialized palliative home care services submit a core data set of routine patient information, including demographic data, principal diagnoses, symptoms, outcomes of palliative care, and service-associated data in the context of care pathways (referring institutions, treatment post-discharge). The HOPE Symptom and Problem Checklist (HOPE-SP-CL) for palliative care patients was developed from a survey in 2001 that identified common physical, nursing, psychological, and social issues through free-text entries. This checklist includes 16 items covering eight physical symptoms, two nursing topics, four psychological issues, and two social problems, with space for additional notes. It employs a four-step verbal rating scale for symptom intensity - differentiating no, mild, moderate, and severe - and is filled out by healthcare staff at both admission and discharge or death, not by patients themselves, to supplement existing self-assessment tools [16, 17].

The data analyzed related to palliative care settings as follows: In Germany, *inpatient palliative care wards* are usually located within general hospitals and admit patients in crisis and medical instability. *Inpatient hospices* are free-standing services providing end-of-life care for patients who cannot be cared for at home. Clinical hospital staff, patients and their families may involve *hospital palliative care support teams* for specialist advice and support. *Volunteer hospice services* and *outpatient hospice and counseling services* provide psychosocial and emotional support, networking and befriending to patients living at home, in nursing homes or inpatient hospices. *Specialized palliative home care* teams are multiprofessional teams providing additional specialized palliative care in patients’ homes, nursing homes or inpatient hospices [18].

A concomitant of the register’s voluntary character is the in-homogeneity of the data submitted by different facilities and types of setting. It is expectable that the register does not include all patients receiving palliative care; the proportion of cases in the population that the register covers varies according to the type of setting in question. Data from *N* = 69,116 patients aged over 64 were available. The mean age of the cohort was 80.99 years (SD 8.82); half the sample was male; the majority of patients had a principal diagnosis of cancer (64.8%; *n* = 44,758).

### Statistical analysis

We ran descriptive and inferential statistical analysis with SPSS Statistics (version 28.0.0). The data on age, sex and year of care provision were complete (*N* = 69,116). Patients without a documented principal diagnosis of dementia were treated in the analyses as not having dementia, although it is possible that dementia was an additional or supplementary diagnosis in these cases. Where there was no record of the palliative care institution that had submitted the register entry, we subsumed the case in the “other palliative care” category (*N* = 72; 0,1%). Similarly, cases with unspecified palliative care setting as referring institutions were categorized under “other providers” (*N* = 7,460; 10%), equivalent handling with discharging institutions (*N* = 2,116; 3,0%). Missing data on the outcome of palliative care fell into the category “other outcome” (*N* = 81; 0,1%).

To reduce instances of missing data, we combined the diagnoses documented at admission and discharge. Nevertheless, 4,542 (6.6%) cases were without entries in the diagnosis variable. In about 7% of all cases, the diagnosis was not available as an ICD-10 code, but rather verbatim. The verbatim diagnoses were then translated into ICD-10 codes where this was clearly possible. We categorized the diagnoses in four groups: “dementia”, “cancer”, “non-cancer diagnosis excepting dementia”, and “other documented diagnosis,” such as a symptom or the state or condition of the patient at the time of documentation (e.g. ICD-10 R).

For purposes of sensitivity analysis, we treated missing data on the single symptom “disorientation/confusion” at the beginning of treatment for the analysis as if the symptom had been categorized as not present. Missing data on the symptom item were evident in 45% of the cases at treatment start, but data at discharge on the symptom were complete. Because of the high proportion of missings on that item at treatment start, we additionally present a complete case analysis and an analysis of the discharge item in the sensitivity analysis section.

### Validity check/sensitivity analysis

We performed sensitivity analysis by exploring indications on the validity of dementia diagnoses as recorded in the register. We compared the prevalence of the recorded single symptom “disorientation/confusion” between people with and without a documented dementia diagnosis. In several studies [19, 20] disorientation is stated as one possible cognitive symptom of dementia which may lead in consequence to behavioral or psychological symptoms of dementia (BPSD). Confusion, in contrast is seen as one possible symptom in the range of BPSD. We compared the prevalence of coded “disorientation/confusion” at admission with the following hypothesis:

- We expect the percentage of patients without a documented dementia diagnosis recorded as showing disorientation/confusion on admission to be lower than the equivalent proportion of documented dementia patients.

The confirmation of sensitivity hypothesis with significant inferential statistical findings may be indicative of the validity of diagnosis recording in palliative care institutions reporting to the register.

## Results

### Prevalence of dementia diagnoses: aims (a) and (b)

The prevalence of documented dementia diagnoses was 3.3% (*n* = 2,265) in the total sample from the register, across all settings. This prevalence varied substantially between types of setting (Table 1).

**Table 1:** Prevalence of dementia diagnoses (F00-F03; G30) in different palliative care settings as documented in the register

Care setting	*N* in this setting	*N* with F00-F03; G30	% of patients in the setting
Specialized palliative home care	46,803	2,014	4.3
Inpatient palliative care ward	19,161	148	0.8
Hospital palliative care support teams	2,380	52	2.2
Volunteer hospice service	621	51	8.2
Inpatient hospice	79	0	0
Other palliative care setting	72	0	0
Total	69,116	2,265	

Data from 2,265 patients with a documented principal diagnosis of dementia are available. The analyses detailed below refer to this subgroup. In relation to research aim (c), we compared this subgroup to palliative care patients without a documented dementia diagnosis (*n* = 66,851).

### Differences in frequency of palliative care setting type among patients with and without documented dementia diagnoses: aim (c)

Palliative patients with dementia mostly appear to avail of palliative care in specialized home care settings (88.9% of patients with a dementia diagnosis; *n* = 2,014). Far fewer of them receive palliative care in specialist inpatient palliative care wards (6.5%; *n* = 148) or, from hospital palliative care support teams (2.3%; *n* = 52). The percentage of patients with dementia diagnoses cared for by volunteer hospice services is similarly small (2.3%, *n* = 51). No patients with dementia recorded in the register between 2009 and 2021 received palliative care in an inpatient hospice or from “other palliative care services”.

The data available suggest that it is mainly specialized palliative home care teams that submit information on palliative patients without documented dementia diagnoses to the register (67.0% of the group of patients without a principal dementia diagnosis; *n* = 44,789). These patients have a four times higher chance to be cared for on inpatient palliative care wards than dementia patients (28.4%; *n* = 19,013 vs. 6.5%). As is the case for people with dementia, hospital palliative care support teams (3.5%; *n* = 2,328) account for a much lower percentage on the register. The share of the group looked after by outpatient hospice services (0.9%; *n* = 570) is smaller still, and inpatient hospices and “other palliative care services” amount to just 0.1% each (*n* = 79 and *n* = 72 respectively) of the palliative care arrangements registered for patients without dementia diagnoses.

### Outcomes of palliative care in patients with and without documented dementia diagnoses: aim (c)

Palliative treatment ended in death for patients with documented dementia diagnoses significantly more often than was the case for patients without dementia diagnoses; transfer to other care settings (including other palliative settings) or discharge to their place of residence was significantly more frequent for the latter group (χ² = 161.898; *p* < .001). This significance persisted after Bonferroni correction of the alpha level. Stabilization of the patient’s condition, a rare outcome overall, occurred twice as often in patients with documented dementia than in those without this diagnosis (Table 2).

**Table 2:** Comparison of palliative care outcomes in patients with and without a dementia diagnosis

Outcome	*N* (dementia)	% (dementia)	*N* (no dementia)	% (no dementia)
Deceased	1,666	73.6	42,870	64.1
Discharge / transfer	450	19.9	20,440	30.6
Condition stabilized	58	2.6	883	1.3
Other	91	4.0	2,658	3.9
Total	2,265		66,851	

### Care pathways of patients with and without documented dementia diagnoses: aim (c)

General practitioners were the most frequent source of referrals to palliative care for both patients with a documented dementia diagnosis (47.7% of this group; *n* = 1,081) and those without (39.9%; *n* = 26,655) (see Table 3). While more than a quarter of patients with a dementia diagnosis were referred by a trained/qualified palliative care physician (26.3%; *n* = 595), the same was the case for only 14.6% (*n* = 9767) of non-dementia patients. About ¼ of patients with dementia had been directly transferred from nursing homes to palliative care settings (23.7%; *n* = 536); by contrast, nursing homes account for a much smaller percentage of patients without dementia (7.0%; *n* = 4,692). A significant proportion of the non-dementia group had been referred directly from an inpatient stay in a general hospital (18.3%; *n* = 12,230).

**Table 3:** Care pathways of patients with and without a documented dementia diagnosis: healthcare provider(s) that referred the patient to palliative care (multiple responses possible)

Referring institution/service	*N* (dementia)	% (dementia)	*N* (no dementia)	% (no dementia)
General hospital	150	6.6	12,230	18.3
Nursing home	536	23.7	4,692	7.0
General practitioner	1,081	47.7	26,655	39.9
Home care (general)	299	13.2	8,135	12.2
Home care (providing palliative care services)	116	5.1	3,324	5.0
Ambulatory health care center (MVZ)^a^	2	0.1	105	0.2
Trained/qualified palliative care physician	595	26.3	9,767	14.6
Volunteer hospice service	117	5.2	2,167	3.2
Outpatient hospice and counseling service	7	0.3	510	0.8
Specialized palliative home care service	425	18.8	10,771	16.1
Inpatient palliative care ward (hospital)	26	1.1	4,458	6.7
Hospital palliative care support team	6	0.3	385	0.6
Inpatient hospice	6	0.3	691	1.0
Other provider	438	19.3	7,022	10.5

^a^ MVZ: *Medizinisches Versorgungszentrum*

For obvious reasons, analysis of care pathways subsequent to treatment in the documenting institution or service was restricted to patients who did not die. The available subsamples were *n* = 586 patients with (97.8% of survivors in this group) and *n* = 22,956 patients without documented dementia (95.7% of survivors in this group).

For both groups, discharge to place of residence was the most frequent course of action (Table 4) (29.9% of dementia patients, *n* = 175, and 28.8% of non-dementia patients, *n* = 6,602). Substantial proportions of patients with dementia diagnoses were discharged to a nursing home (17.1%; *n* = 100) or to the care of a trained/qualified palliative care physician (15.7%; *n* = 92). A lot more patients without dementia experienced discharge to general hospitals (17.2%; *n* = 3,957) or to an inpatient palliative care ward (10.1%; *n* = 2,313). It appears, a greater proportion of these patients needed inpatient treatment for their conditions than patients with dementia.

**Table 4:** Care pathways of non-deceased patients with and without a documented dementia diagnosis: discharge to healthcare provider after palliative care (multiple responses possible)

Institution/service to which patient was discharged	*N* (dementia)	% (dementia)	*N* (no dementia)	% (no dementia)
General hospital	57	9.7	3,957	17.2
Nursing home	100	17.1	1,434	6.2
General practitioner	175	29.9	6,602	28.8
Home care (general)	55	9.4	1,886	8.2
Home care (providing palliative care services)^b^	14	2.4	841	3.7
Ambulatory health care center (MVZ) ^a^	0	0	31	0.1
Trained/qualified palliative care physician^b, d^	92	15.7	2,064	9.0
Volunteer hospice service	15	2.6	408	1.8
Outpatient hospice and counseling services ^b, c^	0	0	327	1.4
Specialized palliative home care service^b^	38	6.5	2,481	10.8
Inpatient palliative care ward (hospital)^b^	17	2.9	2,313	10.1
Hospital palliative care support team^b^	4	0.7	254	1.1
Inpatient hospice^b^	2	0.3	1,294	5.6
Other provider	96	16.4	2,006	8.7

^a^ MVZ: *Medizinisches Versorgungszentrum*

^b^ classed as providing a palliative treatment service

^c^ *Ambulante Hospiz- und Palliativberatungsdienste*

^d^ Either a qualified palliative care specialist (*qualifizierter Palliativarzt*) or a physician who has completed training in palliative medicine (*Arzt mit Zusatzweiterbildung Palliativmedizin*)

### Continuity of palliative care

Comparison of surviving patients who received continuing palliative care, found that less than half (42.7%; *n* = 250) of the dementia patients experienced such continuity, contrasting with more than half of those without dementia diagnosis (58.3%; *n* = 13,393). The latter group therefore appears to have a higher chance of continuing palliative care. This difference remained statistically significant after Bonferroni correction of the alpha level (χ² = 57.653; *p* < .001). Table 4 indicates providers we classed as supplying palliative treatment services.

### Sensitivity analysis

At the onset of palliative care, the register data explicitly recorded the symptom “disorientation/confusion” in the case of almost 1/3 of all included patients with a documented dementia diagnosis, but only for one in ten of all included patients without a dementia diagnosis (Table 5) displaying a statistically significant difference (*p* < .001). Looking only at the complete cases concerning documentation of this symptom 18.8% of the 36,700 non-dementia patients and 51.8% of the 1,309 dementia patients displayed confusion/orientation with a statistical difference of *p* < .001. The hypothesis of the sensitivity analysis was confirmed. This is also supported by the comparison of the symptom prevalence at discharge, where no missings in symptom documentation were evident (*n* = 6,367; 9.5% of non-dementia cases; *n* = 480; 21.2% of dementia cases).

**Table 5:** Recorded “disorientation/confusion” in patients with and without a documented dementia diagnosis on admission to palliative care

Point of time	Dementia: *n* (%) with symptom	Non-dementia: *n* (%) with symptom	Intergroup difference (χ²-test)	
			Χ²	*p*-value
Admission (at least moderate confusion/disorientation)	678 (29.9)	6889 (10.3)	865.575	< 0.001
Total sample analyzed	2,265	66,851		

## Discussion

### Principal findings/results of the study

We found that only 3.3% of patients whose data are included in the German National Hospice and Palliative Care Register have a coded principal diagnosis of dementia. The prevalence of this diagnosis varies according to the service that registered the data. On inpatient palliative care wards the prevalence of documented dementia was 0.8%, while hospital palliative care support teams registered a rate of 2.2% and specialized palliative home care services reported 4.3%. These numbers are lower than the prevalence of dementia in Germany’s general population aged 65 and older of 8.6% [21], and lower compared to the prevalence in older hospitalized individuals which was reported by a systematic review between 12.9 and 63.0% [22]. It is also lower compared with the population-prevalence of dementia in international populations, which is largely comparable to that in Germany: 7.1% in the UK’s 65 + population [23]; a meta-analysis of European population studies reveals a prevalence of 5.05% [24]; the 2021 WHO report estimates an European prevalence of 8.46% in people older than 64 years [25]; a worldwide meta-analysis reports prevalence of 7.26% among people in institutional and community settings [26].

### What this study adds

Despite the difference in comparison to the general population, our findings are in line with other national and international studies on where patients with dementia die. A recent German study noted that nobody within a sample of people with dementia had died with inpatient or palliative home care support [7] Research conducted in London found that few people with dementia living in nursing homes or in their own homes received care from a palliative care specialist [27]. Eisenmann et al. state that “death in hospice facilities or palliative care units is very rare […]” [28]. among people with dementia. A five-year cohort study from Taiwan found 1.64% of patients with dementia to receive palliative care [29]. Figures from Belgium indicate that almost 20% of patients with dementia living in nursing homes were admitted to hospital in the last month of their lives, but none of these admissions were for palliative treatment purposes [30]. Ryan et al. (2012) report that people with dementia experience various barriers to accessing palliative care, including failures in awareness or assessment of this group’s needs concerning end-of-life care [31]. Combining these findings with Mo et al. (2021) results on a lacking consensus regarding referral criteria for specialist palliative care in patients with dementia [32] we might consider the existence of barriers to palliative access for people with dementia to be evident. A systematic review by Erel et al. (2017) assessed the barriers that were associated with implementing palliative care for dementia patients [33]. The authors group their findings in four major obstacles: administrative/policy issues (for example financial reimbursement), lack of education (e.g. lack of awareness of palliative care or prognostic uncertainty), deficits in communication (for example between healthcare providers) and reasons relating to staff characteristics (e.g. staffing shortage, time constraints but also attitudes, beliefs and values). Another integrative review, also with the goal to identify limitations that hinder the introduction of palliative care for people with dementia, Santos (2018), came up with mainly similar results: “lack of knowledge, unpredictability of the disease, lack of criteria for indications, lack of communication, limited access and resources, beliefs and preconceptions in relation to death, and refusal by the patient and family” were stated as the main barriers [34].

We had expected higher rates of patients with dementia in specialized palliative home care. It is known that hospitalization can cause negative impacts on people with dementia, which it is good practice to avoid if possible [13, 35]. Thus, the higher proportion of people with dementia in home care as opposed to hospital settings could be indicative of care meeting their needs. Nevertheless, even this number falls far short of the percentage we might expect in view of dementia prevalence in the general population. A possible problem could be lacking access due to several barriers or even denied access. We should although consider the possibility that some individuals with dementia in palliative care might not have had dementia listed as their principal diagnosis, or possibly, and that some may have been undiagnosed. Our current dataset does not allow us to determine how widespread this issue might be, and existing research predominantly focuses on principal diagnoses [36]. Given this, evaluating our study’s findings, including the 3.3% prevalence rate, becomes challenging. This figure might warrant adjustment—potentially higher—based on factors not fully explored in our data, such as the presence of symptoms or comorbidities that necessitate specialized palliative care. Understanding whether these patients exhibited specific symptoms or had other serious conditions like cancer or heart failure could clarify the suitability and timing of palliative care interventions. In most instances, study populations in palliative research are divided into cancer and non-cancer patients [37, 38]. To reduce the risk of underreporting, registers of palliative care users should include a mandatory variable on diagnosis of dementia or cognitive impairment.

### Strengths and limitations of the study

This paper’s results are based on registry data. A large data set of almost 70,000 cases, reported by various palliative care settings in Germany and covering a period of over 10 years, was available; this size represents a strength of our study.

The voluntary basis on which our data was reported to the register limits the findings’ validity. Some settings, such as inpatient hospices, are underrepresented. The number of data entries for this setting is clearly lower than we might expect in view of how many inpatient hospices exist in Germany. Data protection procedures cause that we do not know which institution added which number of data sets to the register. Further, the register records only the patients’ principal diagnosis; some patients may have had an additional or secondary diagnosis of dementia, but this information was not available to our analysis. Additionally, the entry format was inconsistent and verbatim diagnoses had to be transferred to ICD10 codes manually, which can be prone to errors. We mitigated the risk by having it done by two physicians. The use of the single item on disorientation/confusion causes limitations, as it was not possible to match the dementia patients and others according their stage of disease trajectory and other parameters to compare the trajectory of disorientation/confusion throughout treatment. It is probable, that the presented prevalences are underestimating the true prevalence since only those diagnoses/symptoms could be counted that were explicitly documented and there was a relatively high number of missings in the symptom item for sensitivity analysis and more than 6% cases with a missing diagnosis. Some diagnoses were coded with an ‘R’ code in accordance with the ICD system, indicating symptoms or the state or condition of a patient at the time of documentation. This practice is in line with the overall approach of palliative care, which aims to improve patients’ wellbeing and alleviate their symptoms rather than attempting to curatively treat the disease. In these instances, the ‘R’ code, in the palliative care context, effectively acts as the principal diagnosis; it is possible that some of these patients may have had a principal diagnosis of dementia that the palliative care setting did not record.

## Conclusions

The prevalence of dementia according to data from the German National Hospice and Palliative Care Register is lower than the prevalence of dementia in the general hospital population of 65-year-olds and older and lower than the prevalence of dementia reported for the age-comparable general population. However, results are in line with international studies on proportion of dementia patients receiving palliative care. Future research might seek to ascertain whether the small proportion of dementia patients in palliative care records in Germany arises either from dementia not being coded as patients’ principal diagnosis respectively from lapses in documentation after diagnosis of dementia, or indeed from dementia patients experiencing barriers to accessing these services or even from being excluded or triaged out of receiving palliative care.

### Electronic supplementary material

Below is the link to the electronic supplementary material.

Supplementary Material 1

## Data Availability

The data can be requested by contacting the German National Hospice and Palliative Care Register via its website: https://www.dgpalliativmedizin.de/projekte/hospiz-und-palliativregister.html or by email: dgp@dgpalliativmedizin.de.
